# Modelling Vesicular Release at Hippocampal Synapses

**DOI:** 10.1371/journal.pcbi.1000983

**Published:** 2010-11-11

**Authors:** Suhita Nadkarni, Thomas M. Bartol, Terrence J. Sejnowski, Herbert Levine

**Affiliations:** 1 Center for Theoretical Biological Physics, University of California at San Diego, La Jolla, California, United States of America; 2 Howard Hughes Medical Institute, Salk Institute for Biological Studies, La Jolla, California, United States of America; 3 Division of Biological Sciences, University of California at San Diego, La Jolla, California, United States of America; University of California San Diego, United States of America

## Abstract

We study local calcium dynamics leading to a vesicle fusion in a stochastic, and spatially explicit, biophysical model of the CA3-CA1 presynaptic bouton. The kinetic model for vesicle release has two calcium sensors, a sensor for fast synchronous release that lasts a few tens of milliseconds and a separate sensor for slow asynchronous release that lasts a few hundred milliseconds. A wide range of data can be accounted for consistently only when a refractory period lasting a few milliseconds between releases is included. The inclusion of a second sensor for asynchronous release with a slow unbinding site, and thereby a long memory, affects short-term plasticity by facilitating release. Our simulations also reveal a third time scale of vesicle release that is correlated with the stimulus and is distinct from the fast and the slow releases. In these detailed Monte Carlo simulations all three time scales of vesicle release are insensitive to the spatial details of the synaptic ultrastructure. Furthermore, our simulations allow us to identify features of synaptic transmission that are universal and those that are modulated by structure.

## Author Summary

Chemical synaptic transmission in neurons takes place when a neurotransmitter released from a nerve terminal of the presynaptic neuron signals to the postsynaptic neuron that an event has occurred. The goal of our research was to model the release at a type of synapse found in the hippocampus, a part of the brain that is involved with learning and memory. The synapse model was simulated in a computer that kept track of all of the important molecules in the nerve terminal. The model led to a better understanding of the extant experimental data including exact conditions that lead to the release of a single packet of neurotransmitter. According to our model, the release of more than one packet can be triggered by a single presynaptic event but the packets are released one at a time. Furthermore, we uncovered the mechanisms underlying an extremely fast form of release that had not been previously studied. The model made predictions for other properties of the synapse that can be tested experimentally. A better understanding of how the normal synapses in the hippocampus work will help us to better understand what goes wrong with synapses in mental disorders such as depression and schizophrenia.

## Introduction

The synapse from the Schaffer collateral of CA3 pyramidal cells onto CA1 neurons in the hippocampus has been studied extensively due to its role in learning and memory[1]–[3]. These synapses are quite small, and typically contain only one or two “active” zones, specialized regions of the pre-synaptic membrane where vesicles can bind and release their neurotransmitter cargo. Release from these vesicles is governed by the intracellular calcium concentration [Ca^2+^] in the pre-synaptic “bouton”; this is in turn controlled by the local electric potential, via the presence of voltage-dependent calcium channels (VDCC's), which allow for the influx of calcium ions if the bouton membrane becomes depolarized.

Because of its small size and lack of active zone redundancy, hippocampal vesicular release is a highly stochastic process. The most basic feature is the release time course, in units of probability of release per unit time, after a single depolarization. Measurements of this time course have revealed several interesting features. First, the data reveal the existence of multiple time scales involved in this release [1]. This has led to the notion of synchronous release (occurring with only a slight delay after the depolarization) versus asynchronous release (lasting for 100′s of milliseconds). Surprisingly, these time scales appear to be independent of the absolute probability of release p_r_ (i.e. the overall probability that at least one vesicle was released; this is not the same as the *individual vesicle* release probability), even though this probability can vary over a wide range (20% to almost 100%). Exactly why this occurs has not yet been understood.

Furthermore, some experiments have found evidence of a short refractory time (∼ 5–7 msec) after single vesicle release, a time during which additional release is precluded [4]–[6]. Existence of such a refractory period would immediately imply that releases of separate vesicles are not independent, and instead are coupled through either the cell membrane or via specialized proteins in the active zone [7]. Clarifying the extent to which experimental data supports the refractory period concept is crucial, as this result would offer insight into the biophysical mechanisms involved in actual vesicle fusion.

Here, we construct a stochastic spatially-explicit computational model that enables us to realistically simulate the intracellular calcium dynamics in the presynaptic bouton, tracking in detail the progression from depolarization to vesicle release. To do this, we will rely on known ultrastructural details of the CA3-CA1 synapse and also on recent ideas regarding calcium sensor proteins that control the release machinery. The model can be validated with existing release time course data and will be used to address the issues sketched above. We will also consider the effects of genetically knocking out parts of the calcium sensor. Future work will discuss how structural information regarding synaptic geometry and synaptic components can be inferred by combining this model with new measurements.

As will be seen below, our model leads to several important findings. First, we show that in general vesicle release occurs with three distinct timescales. Aside from the fastest one, which is directly controlled by the calcium profile, the other timescales are determined by the sensor kinetics and hence are almost independent of the detailed synaptic geometry. Next, we show that the aforementioned notion of a refractory period is necessary for explaining release data at high probability synapses. Finally, we demonstrate the role of asynchronous release in modulating short-term plasticity. These results help make sense of existing disparate data as well as offer specific predictions for future experiments on hippocampal synapses.

### Model Construction

Exocytosis, the process by which vesicles bind to the membrane and release their neurotransmitter cargo, is primarily triggered by the VDCC calcium currents. The arrival of an axonal action potential (See Fig. 3 in Text S1for the voltage waveform) leads to a depolarization of the membrane potential in the presynaptic terminal and leads to the stochastic opening of VDCCs. The total calcium flux entering the terminal depends on the time course of the action potential, the number of channels present on the membrane, the calcium conductance of open channels, and the total time each of the channels remains open. The calcium ions diffuse away from their point of entry into the terminal, where they may encounter and bind to buffers such as Calbindin, the calcium sensors and the PMCA pumps. A vesicle release takes place if sufficient calcium ions bind to the calcium sensor enabling the sensor to transition into an appropriate active state. The geometrical arrangement of the parts of the calcium handling machinery and the calcium flux entering the pre-synaptic terminal tightly regulate the local calcium profile at the active zone and therefore control the neurotransmitter release probabilities.

**Figure 1:**
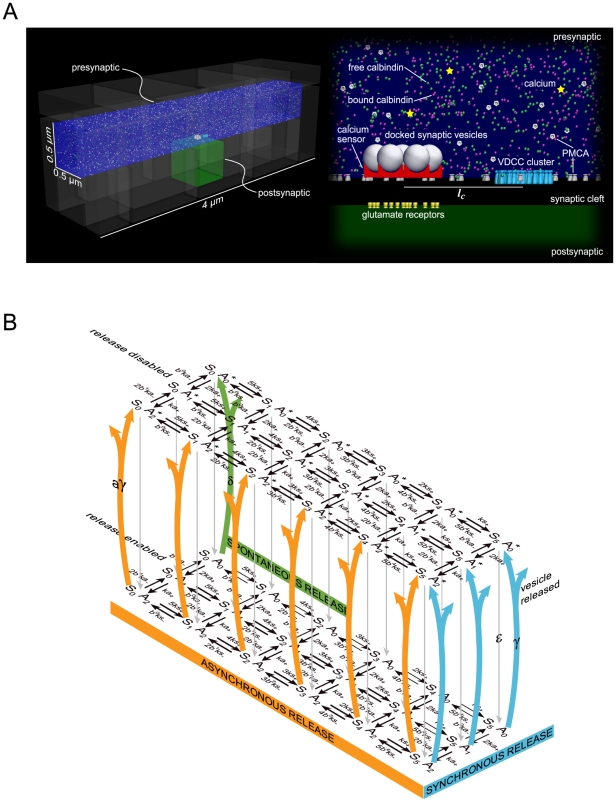
Canonical CA3-CA1 synapse. (**A**) The model Shaffer collateral axon (blue) from CA3 making an en passant bouton (green) with the dendrite of a CA1 pyramidal neuron showing (right) the physiological spatial distributions and concentrations of ligands and molecules. The simulations were carried out in 0.5 µm×0.5 µm×4 µm volume of the axon including of a cluster of voltage dependent calcium channels (VDCCs), mobile calcium buffer calbindin and plasma membrane calcium ATPase (PMCA) pumps. The active zone was populated by seven docked vesicles each with its own calcium sensor for neurotransmitter release at a prescribed distance, *l_c_* from the VDCC cluster. (**B**) Kinetic model for the calcium sensor with 2 pathways, synchronous and asynchronous. The synchronous release pathway has five calcium binding sites whereas asynchronous release has two calcium binding sites. Note that the neurotransmitter release process has distinct rates, *γ,* for synchronous release and a slower one, *aγ*, for asynchronous release. When the refractory period was implemented, the release machinery was disabled after a release event takes place, whether via either synchronous or asynchronous, and was re-enabled with a time constant, ε, of 6.34 ms.

The canonical CA3-CA1 en passant synapse geometry used in our simulations is shown in Fig. 1A. The basic computational domain consists of a pre-synaptic terminal (a bouton) encompassing a rectangular box 0.5 µm wide and 4 µm long; this terminal represents a segment of axon making an en passant synapse, and the only information passing from axon shaft to bouton is the voltage. The dynamical model for calcium handling consists of (Table 1 for rates accompanied by references) 1. a cluster of voltage-dependent calcium channels (VDCCs) of type P/Q [8], which is known to be the main contributor to presynaptic Ca^2+^ current in mature hippocampal presynaptic terminals [9], [10]; 2. plasma membrane calcium ATPase (PMCA) pumps that work to keep the base level Ca^2+^ at 100 nM ; 3. the mobile calcium buffer calbindin-D28k [11] ; 4. an active zone populated by seven docked vesicles [3],[12], each endowed with its own calcium sensor for neurotransmitter release; and 5. the calcium concentration was clamped at 100 nM at both ends of the axon segment. The active zone is placed at a specified co-localization distance, *l_c_* (center-to-center distance: 20 nm–400 nm) from the VDCC cluster (source of Ca^2+^ flux) [13]. Calcium buffers modify the calcium diffusion rate and ultimately the local calcium profile. The diffusion length for calcium ions in our system was measured over several hundred trials and fit to the diffusion equation to calculate the effective diffusion constant. This was ∼50 µm^2^/s, close to experimentally measured values [14] (compared to the free diffusion constant of ∼220 µm^2^/s in the cytoplasm) and our local calcium profiles compare well with those of other studies [15](See Fig. 1 in Text S1).

**Table 1:** Model parameters.

Parameter [and reference]	Value
Calcium diffusion Constant (D_Ca_) [14]	220 µm^2^/s
Calbindin diffusion constant (D_cb_) [67]	28 µm^2^/s
PMCA diffusion Constant (D_PMCA_)	0 µm^2^/s
Voltage dependent calcium channel (VGCC) diffusion constant (D_vgcc_)	0 µm^2^/s
Glutamate diffusion constant (D_glu_) [68]	200 µm^2^/s
Resting intracellular calcium concentration	100 nM
Intracellular calbindin concentration [69]	45 µM
PMCA surface density[70]	180 µm^−2^
VDCC number [9]	1–208
Distance between the active zone and the VDCC cluster (*l*_c_) [49]	10–400 nm
Location of local Ca^2+^ measurement	10 nm (⊥ distance) from the active zone
Maximum radius of the VDCC cluster	66 nm
**Calbindin-D28k ** [**71**]	
Association rate, high affinity site (kh_+_)	0.55×10^7^ M^−1^ s^−1^
Dissociation rate, high affinity site (kh_-_)	2.6 s^−1^
Association rate, medium affinity (km_+_)	4.35×10^7^ M^−1^ s^−1^
Disassociation rate, medium affinity (km_-_)	35.8 s^−1^
**PMCA ** [**70**]	
Association rate (kpm_1_)	1.5×10^7^ M^−1^ s^−1^
Disasociation rate (kpm_2_)	20 s^−1^
Transition rate 1 (kpm_3_)	20 s^−1^
Transition rate 2 (kpm_4_)	100 s^−1^
Leak rate (kpm_leak_)	12.5 s^−1^
VDCC [8]	a_i_(v) = a_i0_ exp(v/v_i_) and b_i_(v) = b_i0_exp(-v/v_i_)Action potential transient reproduced from [8]
α_10,_ α_20,_ α_30,_ α_40_	4.04, 6.70, 4.39, 17.33 ms^−1^
β_10,_ β_20,_ β_30,_ β_40_	2.88, 6.30, 8.16, 1.84 ms^−1^
v_1,_ v_2,_ v_3,_ v_4_	49.14, 42.08, 55.31, 26.55 mV
**Phenomenological Calcium sensor model for the entire active zone**	
Association rate, synchronous release (ks_+_)	1.91×10^8^ M^−1^s^−1^
Dissociation rate, synchronous release (ks_-_)	7.25×10^3^ s^−1^
Association rate, asynchronous release (ka_+_)	3.68×10^6^ M^−1^s^−1^
Dissociation rate, asynchronous release (ka_-_)	26 s^−1^
b, γ, γ^1^, ε	0.25, 6×10^3^/s, 0.417×10^−3^/s, 6.34 ms
**Calcium sensor model (** **Fig. 1** **)**	
Association rate, synchronous release (ks_-_)	0.612×10^8^ M^−1^s^−1^
Dissociation rate, synchronous release (ks_-_)	2.32×10^3^ s^−1^
Association rate, asynchronous release (ka_+_)	3.82×10^6^ M^−1^s^−1^
Dissociation rate, asynchronous release (ka_-_)	13 s^−1^
b, γ, δ, ε, a	0.25, 2×10^3^ s^−1^, 0.417×10^−3^ s^−1^, 6.34 ms, 0.025

Our basic protocol is to simulate the sequence of events at the CA3-CA1 synapse beginning with the arrival of an action potential, the opening of the VDCC's, the diffusion of calcium from the VDCC's to the calcium sensor and the triggering of vesicle fusion and glutamate release [16]. The dynamics of these events were simulated in 3D using Monte Carlo methods (MCell version 3 – see supplemental info for a description of this package). Because the simulations are stochastic, we perform 10000 trials of each test case to generate an average release profile that can be compared directly to experimental data. A detailed analysis shows that the most important source of stochasticity is the random opening and closing of the VDCC's [17].

Release at a single active zone with seven docked vesicles is governed by a dual calcium sensor kinetic scheme (Fig. 1B). The dual sensor kinetic scheme used in these simulations is similar to that proposed (for a different synapse – see discussion later) by Sun et al. [18], in which one of the sensors regulates synchronous release via Synaptotagmin II (Syt II) and has 5 calcium binding sites, while the other regulates slow, asynchronous release via an as yet unidentified molecule and has 2 calcium binding sites. To fit data from the hippocampal synapse of interest, we have adjusted the asynchronous sensor rate (from its value in ref. [18]) (reduced unbinding rate by a factor of 5). We have investigated other possible binding schemes for the asynchronous sensor (data not shown) and attempts to reproduce the asynchronous release were most successful when 2 binding sites were assumed. The vesicle fusion rate for asynchronous neurotransmitter release was taken as an independent parameter, not necessarily equal to the synchronous vesicle fusion rate; identical fusion rates for both sensors, as in the model of Sun, leads to inconsistencies, as discussed in detail later. We simulated the effects of varying the extracellular calcium concentration on the number of vesicles released (See Fig. 4 in Text S1) in the first 20 ms for direct comparison with [1]. The results fit well with the Dodge and Rahamimoff equations with an exponent of 4. Thus the apparent cooperativity is ∼4 even though there are 5 binding sites. The precise values of all our model parameters are given in the table in the supplementary information.

In our baseline model, simultaneous release of multiple vesicles is prevented by imposing a refractory period of 6 ms after a release event takes place [5], [6]; we also consider a variant with no refractory period, everything else being held constant. Finally, the model includes a readily-releasable pool (RRP) with 7 docked vesicles [3], [12], which is decremented after a release. This feature allows the model to accurately describe plasticity phenomenon such as depression and facilitation. All the results described below unless explicitly stated remain valid for a range of typical RRP sizes (results not shown).

## Results

### Validation

**Figure 2:**
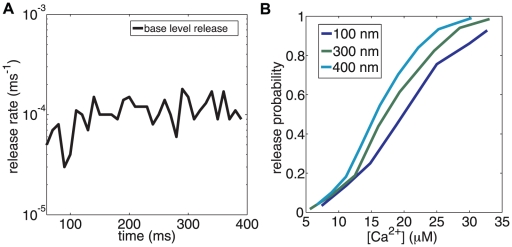
Model validation. (**A**) The neurotransmitter release profile with no external stimulus illustrating the basal release rate. This steady state release profile is a distinct characteristic of the calcium sensor and is independent of geometry. The transient seen in the data is due to starting the simulation off with the sensor in the completely unbound state. (**B**) Calcium sensitivity of neurotransmitter release response for a range of distances, *l_c_* between the calcium sensor and the VDCCs. The VDCC number is adjusted to give the release probability. A set of non-overlapping curves emerge for various distances. Local peak calcium concentration at the site of the active zone is a measure that is modulated by spatial details.

As mentioned above, the calcium is kept at a resting level of 100 nM by the action of the pumps. This resting level gives rise to a base level rate of neurotransmitter release in the absence of any stimulus. This level depends only on the sensitivity of the calcium sensors and not on any of the structural parameters (such as *l_c_* ) which only effect stimulus response. We have verified that the spontaneous release rate in our model (1.2×10^−4^ per ms±0.2×10^−4^ per ms , Fig. 2A) matches the release rate of 10^−5^ to 10^−4^ per ms reported in recordings from CA3-CA1 [19], [20]. This agreement helps validate the values chosen for the forward and backward binding rates of the calcium sensor.

Different hippocampal synapses can have rather different overall probabilities of successful vesicle release. Most hippocampal synapses have a low probability with an average baseline value of p_r_ ∼0.2 [2]. However, the range of release probabilities at hippocampal synapses is high, from weak synapses (*p_r_*<0.05) that rarely ever release to synapses with high release rates (*p_r_*>0.9) [2]. Our model can accommodate this, since the peak value of calcium depends on two distinct parameters; the co-localization distance (*l_c_*) and the number of VDCC's. Fig. 2B shows the neurotransmitter release probability as a function of the peak of the local calcium transient (measured at 10 nm from the sensor) for multiple co-localization distances (*l_c_*). The number of VDCCs present in the cytoplasmic membrane regulates the calcium flux at the specified *l_c_*. Small *l_c_* leads to sharper, narrower local calcium peaks at the active zone (See Fig. 2 in Text S1) and the response curves for different *l_c_* are non-overlapping (Fig. 2B). Our model synapse achieves *p_r_* = 0.20 with 48 VDCCs in a single cluster of 35 nm radius, at *l_c_* = 250 nm, which is compatible with estimates made at other central synapses [13].

**Figure 3:**
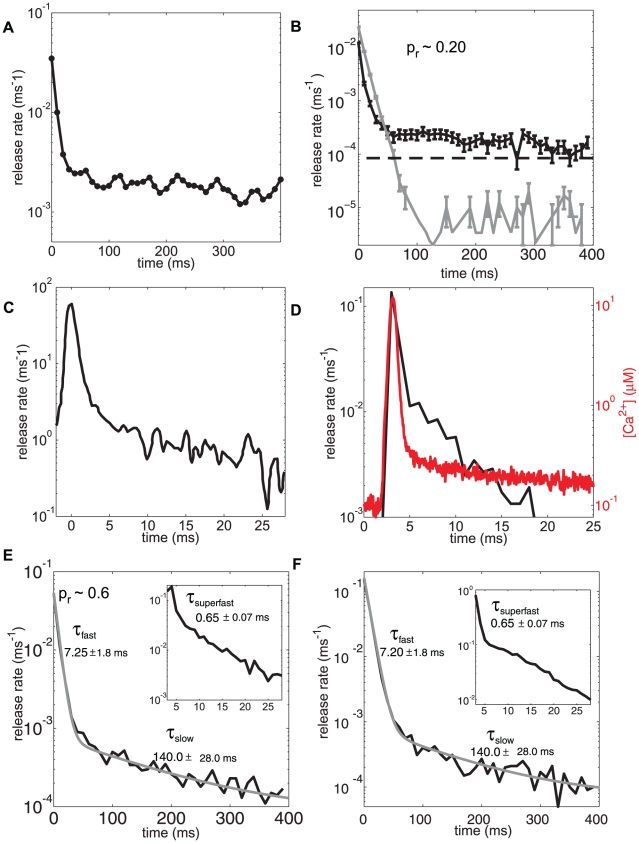
Quantal release time courses. (**A**) Stimulus evoked neurotransmitter release data from dual patch clamp recordings in paired cells using hippocampal pyramidal neurons showing two time scales of release. Figure adapted from Goda and Stevens [1], Fig. 4. (**B**) Black line shows simulation of neurotransmitter release transient for a synapse with intrinsic *p_r_* = 0.2 showing two distinct time scales of release (10 ms bins, compare with 3a). Grey line with shows simulations of kinetic model by Sun et al. [18] in a CA3-CA1 with a single active zone. Dashed grey line describes the average base level (no stimulus) release. (**C**) Figure adapted from from Scheuss et al. [22], Fig. 6. Measured release transient at the calyx of Held showing a fast timescale of release. (**D**) A superfast time scale (*τ_superfast_*) emerges for neurotransmitter release rate (*p_r_* = 0.2) using finer 1 ms bins (left axis, black line). Compare with the superfast timescale of release described at the calyx in 3C. The calcium pulse measured 10 nm from the calcium sensor in response to 48 VDCCs at *l_c_* = 250 nm that triggered neurotransmitter release is superimposed (right axis, red line). The initial superfast part of the release is highly correlated to the calcium pulse (phasic synchronous release) and is followed by a fast timescale of release (delayed synchronous release). (**E, F**). Release transient in response to an action potential for synapses with *p_r_* = 0.6 and *p_r_* = 0.95 in 10 ms bins. The insets show the superfast timescale for the same data (1 ms bins). The release transient for *p_r_* = 0.6 is generated for synapse with 128 VDCCs placed 400 nm from the sensor and 112 VDCCs placed at 250 nm for *p_r_* = 0.95. Even though the maximum amplitudes of the two components of release in a *p_r_*-dependent way, the 3 decay time constants *τ_superfast_*, *τ_fast_* and *τ_slow_* are insensitive across a wide range of release probabilities. The decay time scales are also independent of ultrasynaptic structure (compare b, d, e, f). For a synapse with *p_r_* = 0.2 , 44% of release takes place at *τ_superfast_* , 43% at *τ_fast_*, and the remainder at *τ_slow_*. For comparison to Goda and Stevens [1] exponential decay times scales are fit to the equation a_0_ exp (-t/*τ_fast_*) +a_1_ exp (-t/*τ_slow_*) +a_2._ For **B,** *τ_fast_*  = 6.0±0.7 ms, *τ_slow_*  = 160.0±14.1 ms (a_0_ = 0.025, a_1_ =  0.00023 and a_2_ = 0.00012). For **E,** *τ_fast_*  = 7.0±0.7 ms, *τ_slow_*  = 150.0±14.1 ms (a_0_ = 0.053, a_1_ =  0.00070 and a_2_ = 0.00008). For **F,** *τ_fast_*  = 8.5±0.7 ms, *τ_slow_*  = 120.0±14.1 ms (a_0_ = 0.16, a_1_ =  0.00080 and a_2_ = 0.00007). The ‘superfast’ timescale with 1 ms binning was fit to the equation b_0_ exp (-t/*τ_superfast_*) +b_1_ exp (-t/*τ_fast_*) +b_2_ exp (-t/*τ_slow_*) +b_3_. For **D (inset),** *τ_superfast_*  = 0.7, *τ_fast_*  = 7±0.7 ms *τ_fast_*  = 160.0±14.1 ms (b_0_ = 0.01, b_1_ =  0.0009 and b_2_ = 0.00005 and b_3_ = 0.000015).

In our model, a single action potential at a synapse with 20% release probability produces a roughly 400 msec long elevated release rate of neurotransmitter. The model thus correctly captures the release profile of hippocampal neurons reported by Goda and Stevens [1], adapted figure shown in Fig. 3A. More specifically, the response to an action potential averaged over 10000 trials in 10 ms bins (Fig. 3B, black line, 3E and 3F) gives decay time constants of t_fast_ (7.25±1.8 ms) and t_slow_ (140.0± 28.0 ms) in agreement with the reported data [1], (Fig. 3A). Requiring this agreement enabled us to determine values for the dual sensor model. To show the sensitivity of these results, we have also plotted in Fig. 3B (grey line) the results that would hold for choosing the Sun et al. dual-sensor parameter set (essentially using their sensor kinetic scheme in our spatially-extended simulation) [18]. Clearly, there needed to be an increase in the overall contribution of asynchronous release, as well an increase in the rate of decay of the synchronous release (*t_fast_*). Remarkably, we have been able to accomplish this fit without having to alter the binding affinity of the synchronous pathway, which remains at 38 µM. This affinity is the primary determinant of the calcium sensitivity, since the fast component contributes more than 90% to the overall release probability (Table 1).

### Timescale Results

The first set of issues we address concern a more precise look at the timescales involved in the vesicle response. Fig. 3D (red line) shows the local [Ca^2+^]_i_ 10 nm from the active zone (units on right-hand axis of graph). The neurotransmitter release peaks after a typical latency of ∼3 ms. Note that here we measure the latency starting from the beginning of the action potential (See Fig. 3 in Text S1, i.e. t = 0 in Fig. 3D is at the beginning of the action potential), This latency is due mainly to the delay in opening the VDCCs after the action potential depolarizes the axon. The local [Ca^2+^]_i_ peaks at 12±4.8 µM for p_r_  = 0.2.

This rapid timescale response is present in the vesicular release curves as well. When the data from our standard *p_r_* = 0.20 simulation are binned at 1 ms (Fig. 3D black line, units on left-hand axis), a third “super-fast” timescale of release is apparent. Its time constant, *t_superfast_* = 0.65±0.07 is obviously directly correlated with the aforementioned time course of the Ca^2+^ pulse. This phenomenon arises due to the fact that the vesicle fusion rate *γ* is chosen to be fast enough to track the calcium transient created by the fast P/Q calcium channels; this speed requirement is well within the range of measured release rates [15], [18]. This result has yet to be observed in hippocampal synapses, due to the lack of sufficient data at this temporal resolution; it has however been found in other synapses (see Fig. 3C and later discussion).

**Figure 4:**
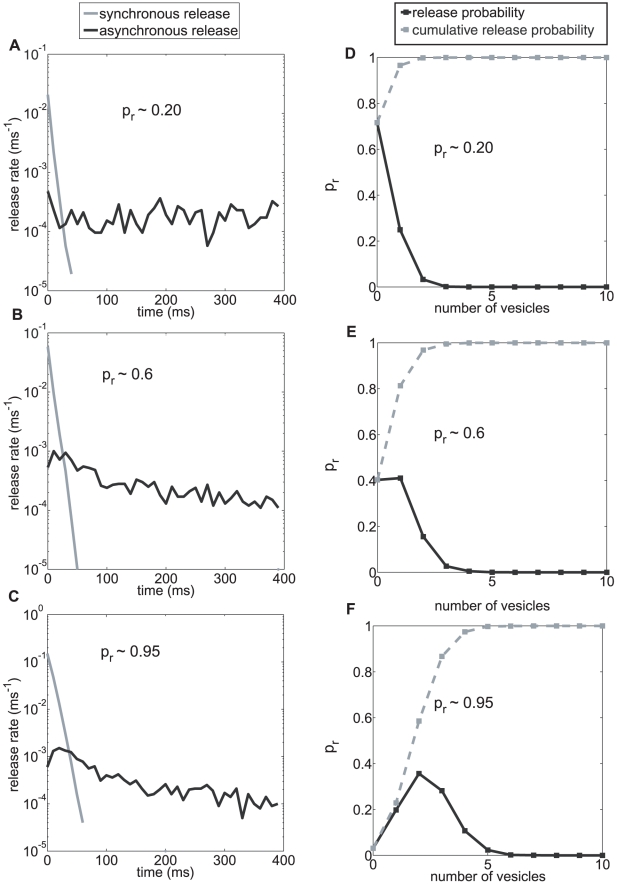
Contributions of synchronous and asynchronous release for a range of probabilities. (**A-C**): The synchronous pathway is the main contributor of the phasic synchronous and delayed synchronous release. The asynchronous release peaks much later. The overall contribution of the asynchronous release increases with release probability (805 events for pr = 0.2, 1213 events for pr  = 0.6 and 1511 events for pr = 0.9). The overall ratio between asynchronous and the first synchronous release however remains small [72]. (**D-F**): The probability distribution (black line) for the number of released vesicles when the RRP is set to be infinite (no depletion after release). Cumulative probability is shown in grey. Consistent with size of the RRP of CA3-CA1, more than 8 vesicles are rarely released. This validates the binding and unbinding rates of calcium ions for the sensor for vesicle release. Also synapses with higher intrinsic *p_r_* are more likely to release more vesicles per stimulus.

The independent contributions of synchronous and asynchronous release are shown in Figs. 4A-C. Initially, the fast (and superfast) release dominates, but it decays rapidly and is soon overtaken by asynchronous release. The synchronous part of the release machinery is the primary contributor to the *t_superfast_* time scale, which should then be referred to as ‘phasic synchronous release’; the *t_fast_* time scale is also mainly driven by the synchronous pathway and is best referred to as ‘delayed synchronous release’; finally, the *t_slow_* release is the commonly named ‘asynchronous release’. The asynchronous contribution to the release profile has a delayed peak compared to the synchronous contribution.

As mentioned above, the model synapse achieves *p_r_* = 0.20 with 48 VDCCs in a single cluster of 35 nm radius, at *l_c_* = 250 nm. This is not unique, since other combinations of VDCC number and *l_c_* can also give *p_r_* = 0.20. Changing the model in this manner does not lead to any significant modification in our findings. What happens if we alter the release probability, by changing either the VDCC number or *l_c_* ? We find that the maximum amplitudes of the synchronous and asynchronous contributions are indeed modulated by the varying *p_r_*, but the decay constants of the release profiles are unchanged (Fig. 3E; *p_r_* = 0.6, *l_c_*  = 400 nm, 128 channels; Fig. 3F; *p_r_* = 0.92, *l_c_*  = 250 nm, 112 channels) . This result of the model is consistent with reported data from high and low release probability synapses that show similar decay constants [1], [21], [22] for the different release probabilities. In other words, in our simulations the decay time scales (other than the super-fast one) are independent of the spatial organization of the synapse and are a consequence of the kinetics of the calcium sensor.

As mentioned above, our model posits that multiple releases can take place from the active zone after a refractory time constant of ∼6 ms following each release [5], [6]. To test the extent to which the finite available resource of docked vesicles (i.e. the RRP) is a limitation, we modify our simulation to contain an active zone in which a released vesicle is instantly replaced, i.e. a depletion free active zone. The probability distribution of number of quanta of neurotransmitter released in 400 ms is shown in Figs. 4D-F. For a synapse with a release probability *p_r_* = 0.2, the likelihood that more than two vesicles are released was less than 5%. Furthermore, there is less than 20% chance of releasing more than 2 and almost never more than 6 vesicles for *p_r_* = 0.6 and a 33% chance of releasing more than 2, and almost never more than 9 vesicles for *p_r_* = 0.95. The size of readily release pool (RRP) has been estimated to be 5–10 vesicles at CA3-CA1 synapses [3]. Thus, the model prediction of the maximum number of vesicles that can be released is consistent with the typical RRP size at this synapse and both these numbers are positively correlated with release probability [23]. The model suggests that the typical RRP size at a CA3-CA1 synapse and the calcium sensitivity of the release machinery are well-matched, so that the number of docked vesicles is not a limiting factor at low stimulus frequencies.

### Refractory Period

Stevens and collaborators introduced the idea that there is a short refractory time following vesicle release from an active zone. With such a refractory period more than one quantum of neurotransmitter can be released by an action potential, but the quanta are released one at a time. Several recent experimental studies have tried to address the question of refractoriness after release but with conflicting results. Explicit measurements at a wide variety of synapses conclude that there exists a “one active zone-one vesicle release” principle and hence provide direct evidence for functional coupling within the active zone [4]–[6], [24]–[31]. However, other studies have presented evidence against uni-vesicular release due to such “lateral inhibition” [4], [32]–[39].

**Figure 5:**
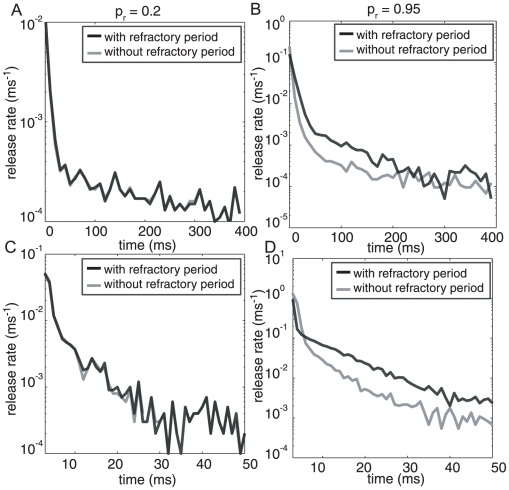
Neurotransmitter release profile for a CA3-CA1 synapse with a single active zone and seven docked vesicles. (**A**) Release data histogram in 10 ms bins for a synapse with intrinsic release probability of p_r_ = 0.2 (48 channels at *l_c_* = 250 nm). Both transient, refractory period transient (grey) and non-refractory period transient (black) almost exactly overlap. (**C**) This holds true for a finer 1 ms bin (bottom panel) as well. (**B**) Release data histogram in 10 ms bins for a high release probability p_r_ = 0.92 (48 channels at *l_c_* = 250 nm). The two transients in this case decay with different rates. The synapse without the refractory period decays faster, as depletion of neurotransmitter vesicles cause decreasing release probability. (**D**) This effect is seen in more detail with 1 ms bins at the same synapse. Only for the synapse with refractory period are the characteristics time scales of decay conserved across the whole range of release probability.

Our basic strategy is to compare neurotransmitter release profiles with and without the existence of a 6 ms refractory time constant preventing simultaneous release of different vesicles. We do this comparison for different values of the overall release probability (See Fig. 5). For a release probability at CA3-CA1 of *p_r_*  = 0.2, the release transient for a synapse with a refractory period (gray line) is almost indistinguishable from a synapse without any refractoriness (black line). Thus for this set of parameters, the presence or absence of refractoriness does not make any functional difference. For a release probability of *p_r_* = 0.2 for the whole active zone, each of the 7 individual docked vesicles must have a release probability of 0.031 so the probability that 2 or more vesicles being released is only 0.02. This implies that although any single vesicle was released on 20% of the stimuli, two or more vesicles were released on only 2% of the trials. The detailed timing of release of the second vesicle relative to the refractory period has a negligible effect on the overall averaged release profile. The consequence of a refractory period was more prominent for *p_r_* = 0.95. For a synapse with independent releases (i.e. no refractory period) and *p_r_* = 0.95, 2 or more vesicles were released on 67% of the trials. The top panel in Fig. 5B shows the release transients over 400 ms when the release data were in 10 ms bins and the bottom panel (Fig. 5D) describes the same data with finer 1 ms bin. Now, there is a clear consequence to the inclusion of a refractory period.

We have seen that our model can reproduce one of the important distinguishing characteristics of neurotransmitter release in hippocampal CA3-CA1 synapses, that the decay time scales are conserved across a wide range of release probabilities even as the overall amplitude of the transient is modulated [21], [22], This result depends on the inclusion of refractoriness. Without refractoriness, depletion overwhelms the release at high release probability synapses: The peak release rate is higher, the decay becomes significantly faster and the amplitude of later releases is much lower (Fig. 3F, black line). We therefore conclude that existing experimental data strongly support the existence of the refractory period.

**Figure 6:**
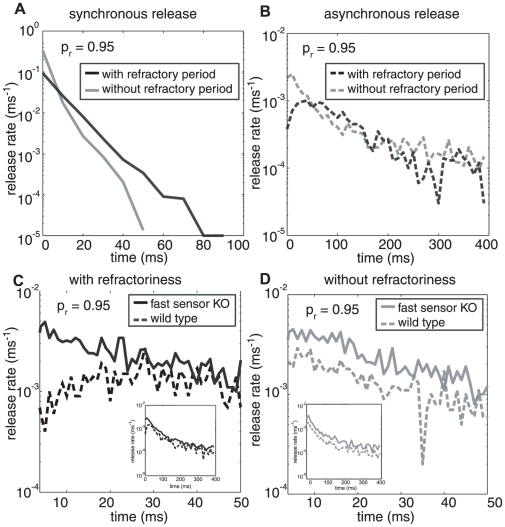
Differences seen due to refractoriness in components of synchronous and asynchronous release. (**A**) For a synapse with refractoriness the synchronous release has a shorter, broader peak than the synapse without refractoriness. (**B**) The asynchronous release channel encompasses more events for synapse with refractoriness compared to without refractoriness. **Neurotransmitter release profile for fast sensor KO and wild type for a synapse with and without refractoriness (1 ms bins).** (**C**) The neurotransmitter release profiles for asynchronous release in wild type and fast sensor KO varieties of the synapse with refractoriness (grey) diverge as they approach shorter time scales of less than 20 ms . Fast release through the synchronous pathway suppresses release from the asynchronous pathway due to the refractory period in the wild type, leading to a dip in asynchronous release. (**D**) The release profiles of wild type and fast sensor KO run almost parallel through the 400 ms transient in the synapse without (black) a refractory period. The transgenic fast sensor KO in both kinds of synapses (with and without refractoriness) is more elevated than the wild type as there is no depletion of vesicles, through the synchronous pathway, from the limited resource available in the RRP. The release starts 3 ms after initiating the action potential (see Fig. S3, as mentioned in the timescale results on page 10) and we have therefore not included this early period in the graphs having 1 ms binning (**C** and **D**).

We can also examine the differences in the release transients due to refractoriness separately for the synchronous and asynchronous release for *p_r_*  = 0.95 (see Fig. 6A and B). This analysis was possible because our sensor model treated these releases via independent pathways (see Fig. 1B). Our model predicts that the synchronous release profile (Fig. 6A) should be lower in amplitude and decay more slowly for a synapse with a refractory period. Synchronous and asynchronous releases compete for the same RRP resources [40] leading to a net increase in asynchronous release (1511 total events in 400 ms, for 10000 trials) for the synapse with refractoriness compared to the synapse without refractoriness (1379 total events in 400 ms) (Fig. 6B). Note that in the first ∼50 ms after the stimulus, when release via the synchronous pathway dominates, refractoriness slows the rate of depletion of the RRP (Fig. 6A). Refractoriness also slows down asynchronous release initially (Fig. 6B). But beyond 50 ms, when asynchronous release begins to dominate, the larger residual RRP (because of slower depletion) in synapses with refractoriness means that the net amount of release via the asynchronous pathway can be larger than in synapses without refractoriness.

Gene knock-out experiments are now routinely used to quantify signaling pathways. Knocking out synaptotagmin (KO), the calcium sensor for neurotransmitter release, eliminates the fast release component of the transient but leaves the slow component intact [18], [41]. We can modify our model to allow for the study of the KO transgenics by removing all the states along the synchronous pathway. Since both pathways used the same resource pool of neurotransmitter [40], knocking out the synchronous release sensor makes more vesicles available for release through the asynchronous release sensor. Augmentation of asynchronous release in genetically modified, fast sensor deficient mice has been previously reported in [42], albeit pointing to a different mechanism. Simulation results for asynchronous release transients comparing synchronous sensor knock-out (KO) and wild type are shown in Fig. 6C and D. The results show that the genetic modification eliminates much of the effect of the refractory period (grey solid line and black solid line respectively) with almost the same number of release events for both in the 400 ms (inset) and 50 ms time windows. The genetic modification has a larger effect on the refractory synapse and is qualitatively more consistent with the aforementioned experimental data.

We can understand this effect in more detail by focusing on the change in time-course brought about by the genetic modification. For a synapse without refractoriness, the ratio (Fig. 6D) between the release rate of the wild type and KO stays constant through the transient; however, for a synapse with refractoriness (Fig. 6C), the model predicts that the ratio between wild type and KO would be larger in the first few milliseconds and then taper off with time. This happens because the large forward binding rate of the synchronous part of the sensor dominates release in the wild type and therefore acts to inhibit asynchronous release; this inhibition occurs through refractoriness that lasts a few milliseconds before the asynchronous channel reaches its normal release rate as defined by the binding kinetics. A 90% increase in release rate of asynchronous release in first 50 ms for synapse with refractoriness in a KO compared to the wild type is seen. While a synapse without refractoriness sees an increase of only 75% in a KO compared to the wild type. In a synapse without refractoriness, synchronous and asynchronous releases are independent and therefore they always occur at their normal rates.

### Stimulus Train Responses

Refractoriness differentially affects synchronous and asynchronous release at early and late times after a single stimulus and this effect is sensitive to the initial release probability (Fig. 5). But what happens during a train of high-frequency stimuli? We performed simulations to predict what might be seen in CA3-CA1 synapses when stimulated at 100 Hz for 200 ms (20 stimuli) and we now examine the results for features that would distinguish between synapses with and without refractoriness. This same stimulus protocol was used in a previous study of a different synapse with many active zones [22] and was found to be sufficient to deplete the RRP. We surmised that such a stimulus might therefore be sufficient to deplete the RRP at our model CA3-CA1 synapse with a single active zone.

**Figure 7:**
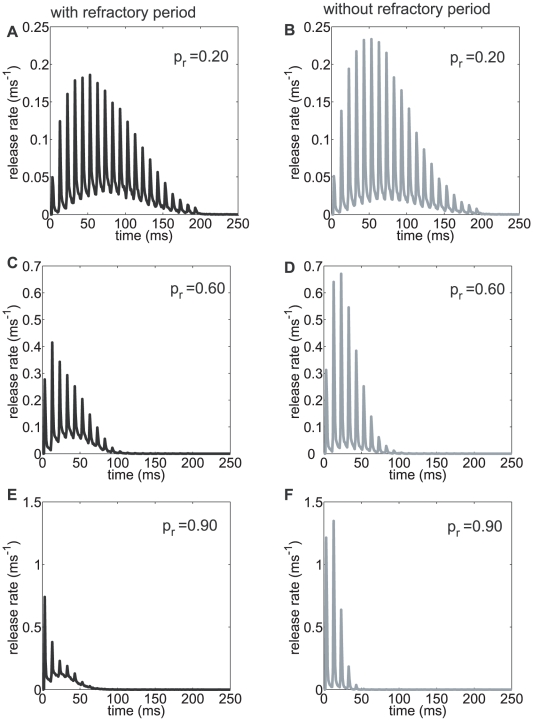
Response to a 200 ms at 50 Hz rate stimulus protocol administered to a model CA3-CA1 synapse with seven docked vesicles. In (**A**) and (**B**) a synapse with low intrinsic release probability of p_r_ = 0.2, in (**C and D**) a synapse with a release probability of p_r_ = 0.6 and in (**E and F**) a high release probability synapse (p_r_ = 0.9) is shown. In the high p_r_ synapse, depletion quickly overwhelms release. Comparing **A** to **B**, **C** to **D** and **E** to **F**, the base level asynchronous release was higher in the synapse with refractoriness (black) whereas the synapse without refractoriness (grey) had higher peak release rates. This is because the refractoriness inhibits immediate release (less that 6 ms interval) from the synchronous pathway and therefore allows the asynchronous release pathway to contribute more to the release. The rates of facilitation and depression were also characteristically different for these synapses.

The response of our model synapse for the different cases of initial release probabilities p_r_  = 0.2 (number of VDCCs  = 48, *l_c_* = 250 nm), p_r_  = 0.6 (number of VDCCs  = 72, *l_c_* = 250 nm), and p_r_  = 0.95 (number of VDCCs  = 112, *l_c_* = 250 nm) is shown in Fig. 7. For p_r_  = 0.6 the facilitation (ratio of first two release rates) in the synapse with refractoriness (black line) was almost twice that of a synapse without refractoriness (grey line). However for the synapse with refractoriness the background release level (due to asynchronous release) was much higher compared to a synapse without refractoriness. These predictions can be directly tested in future hippocampal synapse experiments.

### Short Term Plasticity

**Figure 8:**
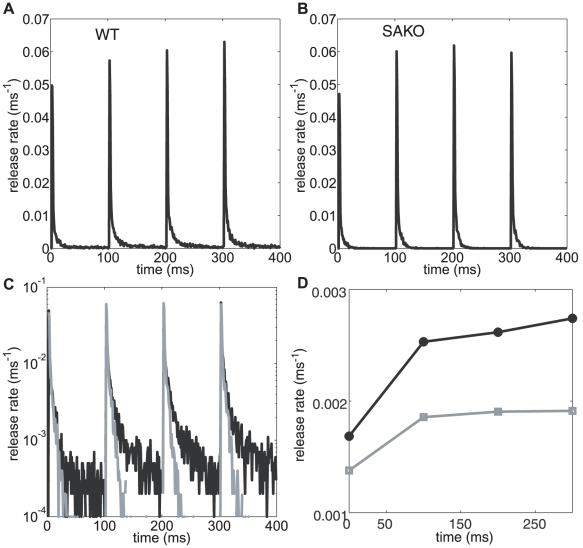
Response to 10 Hz train stimuli. Release rate for wild type (**A**) and a simulated asynchronous release sensor (SAKO) (**B**) plotted in 1 ms bins. The same data is plotted on a log scale to show the elevated long tail of release (black line) due to the presence of asynchronous sensor in the wild type (**C**). The grey line in (**C**) is SAKO. In (**D**) total release rate (100 ms bins) for each stimuli is shown (wild type – black line, SAKO – grey line). The facilitation for the wild-type is 50% as opposed to 35% for the SAKO. In this study vesicle replenishment, which occurs at a timescale of the order of seconds, does not play a role.

We now wish to investigate the role of the slow sensor in the presence of a spike train, the response to a 10 Hz stimuli for a total of 400 ms (i.e. 4 triggers) for a synapse with intrinsic release probability 0.2 is shown in Fig. 8. Response to high frequency 100 Hz stimulus for high release probability synapse is described in the Supporting information (See Fig. 5 in Text S1). The simulations are carried out both for a simulated asynchronous sensor knock out (SAKO) (Fig. 8B) and wild type (Fig. 8A). The response to higher frequency (100 Hz) is discussed in the supplementary material. Unlike the SAKO (Fig. 8B), the peak release rate (data binned in 1 ms) in the wild type (Fig. 8A) is facilitated with each subsequent stimulus. The same data (grey line-SAKO, black line- wild type) is shown on a log scale in Fig. 8C. In the wild type, response to subsequent stimuli rides on top of a higher base level release. This is due to the slow time scale of release of the asynchronous sensor (the inherent memory of the sensor). This ensures greater facilitation for the wild type. Fig. 8D shows the total release rate for each stimulus (grey line-SAKO and black line –wild type). We can see that for the facilitation in the wild type is more than 50% whereas for the SAKO it is limited to 35%.

### Vesicle Fusion Rates

**Figure 9:**
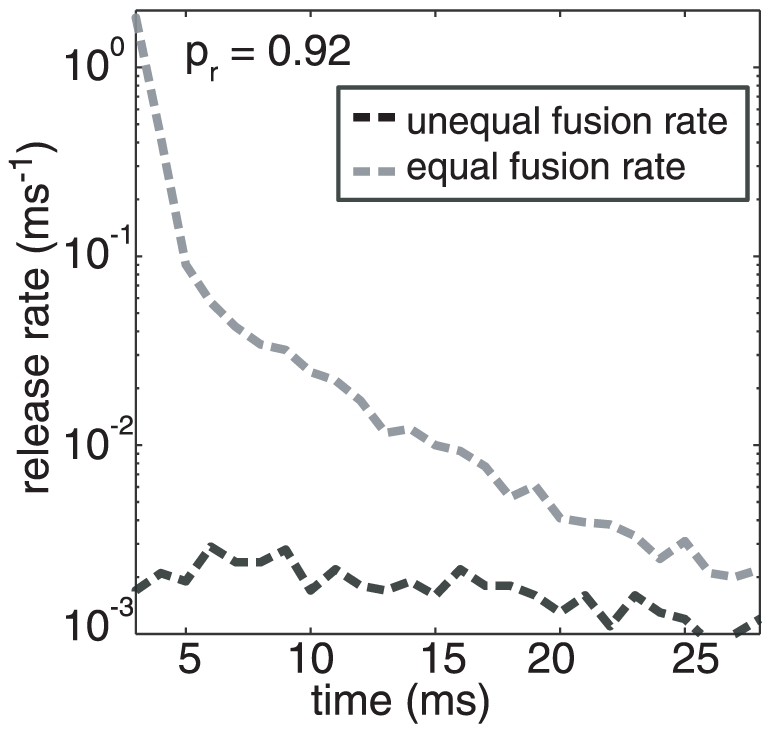
Release profile through the asynchronous pathway with identical vesicle fusion rates for synchronous and asynchronous release, compared with unequal fusion rates (1 ms bins). There is a sharp peak in the asynchronous release after the stimulus that coincides with the calcium signal at the active zone when the vesicle fusion rate is equal for the synchronous and asynchronous case. This peak seen in the simulations is not consistent with observed data. However, slowing down the fusion rate by a factor of 40 matches the data for spontaneous asynchronous release. The X axis starts at 3 ms, this is the delay in release after initiating the action potential (see Fig. S3, as mentioned in the timescale results on page 10).

All the results given so far have used a model for which the parameter *γ_a_*, the fusion rate of vesicles activated by the asynchronous sensor, is smaller than the corresponding rate for the synchronous one. To demonstrate why this is necessary, a sample release profile of the asynchronous pathway for our single active zone synapse with 7 docked vesicles [3] assuming equal release rates for both release pathways is shown in Fig. 9 (Grey line, *p_r_* = 0.2, number of VDCC = 48, *l*_c_ = 250 nm). The early peak in this figure, present for simulations at all values of the release probability, is clearly inconsistent with electrophysiological data [18], [40]. If we demanded equal fusion rates, we were unable to eliminate this early peak in the asynchronous release while still reproducing all the other measured release properties; we tried (to no avail) to accomplish this by changing the binding affinities or by including additional calcium binding sites for the asynchronous pathway that would delay release (data not shown).

Thus, in order for our model to be consistent with measured asynchronous release transients, the value of *γ* needs to be significantly slower for the asynchronous pathway relative to the synchronous pathway. This introduces an additional parameter ‘*a*’ such that the neurotransmitter fusion rate is *γ_a_  = aγ* (with *a*<1) for asynchronous release (see Table 1). The presence or absence of assumed refractoriness does not affect this early peak of the asynchronous pathway. For the choice *a* = 0.025 (i.e. net asynchronous vesicle fusion rate = 50/s), the early release from the asynchronous pathway is suppressed and all the detailed characteristics of neurotransmitter release can be reproduced (Fig. 9, Black line).

In the context of a model with *independent* vesicles comprising the active zone, we must assume that the asynchronous pathway has a slower release. An alternative approach to eliminate the early peak in the asynchronous release while implementing neurotransmitter fusion rates for synchronous and asynchronous release is to use a higher-scale phenomenological model for the entire active zone such that it has a *single* gating mechanism prescribed by kinetic rates given in Table. 1. This type of model sets no *a priori* limit on the number of docked vesicles (i.e. has an infinite RRP) and multiple release events may occur, subject to the refractory time constant. With this framework, it is possible to consistently reproduce all our data, including the 3 timescales and a cumulative release well matched to the RRP (data not shown). In short, an additional parameter ‘*a*’ is needed in the docked vesicle model with individual sensors on each vesicle, to directly suppress asynchronous release, whereas in an alternative phenomenological approach that treats the whole active zone as *having a single gating mechanism*, no such parameter is needed. We have chosen to focus on the individual vesicle model, as there is no obvious justification for such a strong vesicle coupling.

## Discussion

Neurotransmitter release at chemical synapses in response to electrical stimulus is tightly regulated over multiple time scales by mechanisms in the presynaptic terminal. Release takes place at specialized locations at the presynaptic membrane called active zones designated by the presence of SM (Sec1/Munc18-like) proteins [7], [43]. Some of this machinery is ubiquitous for all exocytosis events and consists of SNARE (soluble N-ethylmaleimade-sensitive factor attachment protein receptor) proteins, SM (Sec1/Munc18-like) proteins, along with complexins and synaptotagmins that are needed to control the timing of neurotransmitter release [7], [44]. Much of the molecular and structural details of this process have been elucidated; however, how each of the components interacts to execute precise dynamic control on the release has not yet been established. The goal of this study was to develop a detailed biophysical model of exocytosis that takes into account the spatial organization of the molecular components and the time courses of their kinetic states.

We have chosen to carry out our study focusing on the CA3-CA1 synapse in the hippocampus. The advantage of using this synapse is its relative simplicity, consisting of only one or two active zones, and its starring role in many studies of plasticity. Even with this emphasis, varying results from different experiments have led to confusion regarding certain basic features of synaptic transmission. Our computational experiments have led to possible resolutions for some of these contentious issues, such as the existence of refractoriness between releases, cohesively bring together data from different sources that point to universal features of vesicle release and those that may be unique to the CA3-CA1 synapse [45], [46].

In particular, our simulations have illuminated the observation in two separate sets of data [21], [22] that changing the release probability modifies only the amplitudes of release transients and not the timing of release. An important prediction of this study is the new identification of three separate time scales of the release and that these time scales are all independent of the synaptic geometry. It has been reported in a recent study [47] that properties of the Ca^2+^ channels and relative location of Ca^2+^ do not modulate the relative dynamics of asynchrony to phasic release. This study strongly supports our own modeling results in which the calcium sensor governs all the relevant time scales. This result stands in contrast with other approaches [48] for which geometry governs slow release (see later).

Two decay timescales have indeed been observed in hippocampal synapses. Also, similar findings (a slow decay component of ∼82 ms) have been reported in parvalbumin-containing GABAergic interneurons expressing P/Q calcium channels [21]. However, the predicted super-fast timescale of release has yet to be observed in our hippocampal synapse of interest. It has apparently has been observed in calyx of Held (see later) by Scheuss et al. [22]; see Fig. 3D. Their ‘biphasic decay of release rate’ was comprised of a superfast component of release and a fast component (588.6 ±3.5 µs and 14.7±0.4 ms respectively). However, they were unable to distinguish the contribution of slow asynchronous release lasting up to 200 ms, from the effect of residual glutamate in the cleft. Thus, several different times scales of release by different labs (*τ_fast_* and *τ_slow,_*) or (*τ_superfast_* and *τ_fast_*) have been reported [1], [18], [21], [22]. This disagreement can be reconciled by the coexistence of three time scales of release, as seen in Figs. 3B and 3C.

As has been explained, our model for the calcium sensor is a modified stochastic version of the one introduced by Sun et al. [18]. That kinetic model is one of several that have been created to explain data from the calyx of Held. The calyx of Held is a giant pre-synaptic terminal with hundreds of active zones and can be probed directly because of its large size [49], [50]. However, the active zones are separated from the points of calcium entry (i.e. voltage-dependent calcium channels) over a range of distances. This makes it difficult to disentangle the properties of vesicular release that arise due to the kinetics of the calcium sensors alone from those due to their complex spatial arrangement. Elegant calcium-uncaging experiments have been performed to ensure a uniform calcium concentration across the hundreds of docked vesicles [15], [51]. However, the calcium concentration stays high for a long time in these protocols, depleting the docked vesicle resources and hence modifying the average vesicle release rates. Furthermore, uncertainties in actual number of docked vesicles introduce error in the kinetic models. These difficulties have led to disparate models with calcium sensitivities that vary over 500% [15], [51]. For example Fig. 1 in [13] shows that 25% release probability corresponds to peak calcium of either 8.8 µMor ∼50 µMin two competing kinetic models for the calyx. These models provide a starting point but cannot be directly used to provide an accurate description of release at CA3-CA1.

A detailed comparison of our model for vesicle release and that of Sun et al. is outlined as follows. In contrast to the deterministic kinetic sensor model of Sun et al., our model is a spatially explicit stochastic model of the entire bouton. In Sun et al. [18] the two sensors act completely independently to cause release and all releases are independent events. In our kinetic model for CA3-CA1 the release of one vesicle (whether synchronously or asynchronously) temporarily prevents the release of other vesicles within the active zone. A refractory period results with a recovery time constant of ∼6 ms [5], [6]. Also, our model differs from Sun et al. [18] in the binding and unbinding rates while maintaining the binding affinity and cooperativity of the calcium sensor for synchronous release. To better match published data [1] the asynchronous release in our model lasts much longer and has a much higher amplitude suggesting that this synapse has a longer memory. This was achieved in the model by making the unbinding rate of the second sensor 5 times slower than that in Sun et al. [18]. Another significant distinguishing feature of the present model is that it includes a readily-releasable pool (RRP) with 7 docked vesicles [3], which is decremented after a release.

The calyx and the CA3-CA1 synapses subserve different functions. The calyx is a giant synapse in the auditory pathway that achieves reliable synaptic transmission with several hundred active zones. In comparison, most CA3-CA1 synapses in the hippocampus have an intrinsically low release probability but are highly plastic [23] to serve as a substrate for memory [52], [53]. Despite these differences, the calcium sensor that governs fast temporally correlated signal transmission seems to be conserved. Asynchronous release transients may be more diverse, although at a particular calyx synapse that exhibited an exceptionally high level of asynchronous release, Scheuss et al. [22] reported a slow asynchronous decay with a time scale that was comparable to that in our model (79.3 ±29.7 ms). Furthermore, the global parameters of the synapse, such as the number of active zones, and their respective distance from the VDCCs, can give rise to apparently different calcium sensitivities that can be misleading (see Fig. 2B). In fact, some researchers [48] have attributed the entire mechanism of asynchronous release in the calyx to vesicles that were further away from calcium sources. This is manifestly not the case in our hippocampal model, as we have repeatedly emphasized that the decay time scales were independent of the spatial organization of the synapse and were a consequence of the kinetics of the calcium sensor (See Fig. 3E). Thus is it as yet unclear whether the asynchronous sensor is similar in different synapses. Whether universal or not, a Ca^2+^ sensor with a long memory as described in our hippocampal model can have a significant role in activity-dependent short-term synaptic plasticity (Fig. 8).

We now return to the issue of the refractory period. The active zone is morphologically distinctive and has complex protein meshes spanning the entire length of the region connecting all the vesicles [54]. Recently, a diffusive protein trans-complex was identified that forms a continuous channel lining at the fusion site and is integral to exocytosis [55]. Therefore, it is reasonable to hypothesize that a local perturbation caused by exocytosis is likely to be spread through these diffusive molecules. It has also been suggested that the mechanical rearrangement of the lipid bilayer during exocytosis can also affect later release over a short enough time scale [56]. Given all these opportunities to influence each other, there are likely to be conditions under which docked vesicles interact cooperatively.

Our simulations suggest that the release of a vesicle may trigger direct and indirect interactions between the synchronous and asynchronous release pathways, between individual sensors on the several docked vesicles, and between the microenvironment of the membrane of the active zone and the vesicles. These interactions occur on several time scales. In our model, “Lateral inhibition” a refractory period with a time constant of 5–7 ms [5], [6], [57] blocks simultaneous release from the active zone during the period of highest calcium concentration after opening of VDCCs. The exact biophysical mechanism for this refractory time window is unknown. Without such a refractory period of 6 ms after a release event, it would not be possible to maintain the same decay time scales across all release probabilities (compare *p_r_* = 0.2 and *p_r_* = 0.9 shown in Fig. 5). In addition, the prediction of the facilitation and base level release as illustrated in Fig. 7 can also be rigorously tested experimentally for further confirmation and exploration of the phenomenon.

Some of the discrepancies leading to different conclusions about the refractoriness following vesicle release [4]–[6], [24]–[29], [31]–[37], [39], [58] could be due to differences in techniques and stimulation protocols. The proposed refractoriness originally measured by Dobrunz et al.[5] lasted only a few ms and did not impede subsequent release beyond that time window. Oertner et al. [37] reported multivesicular release accompanied by an increase of glutamate in the synaptic cleft. It is possible that more than one vesicle was indeed released but separated in time by the refractory period, since their methods lacked temporal resolution to resolve millisecond differences. Simultaneous release within synapses containing more than one active zone is also possible [32], [35]. We have estimated that if release indeed operated independently at each docked vesicle, for p_r_ = 0.9 there should be a 70% chance of releasing more than 2 vesicles in response to a single action potential, but in Christie et al. [33] multivesicular release was observed only in a paired pulse facilitation protocol.

The accumulation of glutamate in the synaptic cleft could also give a misleading interpretation of multivesicular release. Abenavoli et al. [34] performed statistical analysis of release events where they observed that the output at long time intervals was not Poisson distributed. This phenomenon was attributed to a burst of release from the same synapse, which contradicted the refractory period hypothesis and led them to conclude that multivesicular release occurred at the CA3-CA1 synapse. An alternative explanation is the existence of long-time correlations in neural activation, perhaps by astrocytes acting to synchronize activity [59], [60]. Furthermore, the quick freeze technique they used to image synaptic vesicles did not have the temporal resolution to distinguish between endocytotic and exocytotic events. In short, we feel that experiments all purporting to see simultaneous release from a single active zone have alternate interpretations.

It has been suggested that synaptotagmins synchronize release rather than control it as an explanation of enhanced asynchronous release seen in transgenic mice with the fast sensor knocked out [42]. Elimination of the fast sensor makes more vesicles available for the asynchronous pathway leading to an augmented asynchronous release in our model. An alternate mechanism has been recently proposed, relying on the molecular zipping action of complexins with synaptotagmins that clamps down release in the wild type [61]. Binding of calcium releases the complexin clamp. However, in the KO this clamp is abolished, leading to an increase in spontaneous release [41]. Further experiments will be needed to test whether this more detailed mechanism is present and important, given that we can already obtain augmentation from the existing model.

Finally, we return to the issue of the universality of fusion rates. Our model has an active zone with a RRP of vesicles that are coupled through a brief refractory period following each release via either the synchronous or asynchronous pathway. This differs from kinetic models for the calyx of Held [15], [51], including that of Sun et al.[18], which assumed that every vesicle release was independent. In the calyx, Sun et al. used a vesicle fusion rate (*γ* = 6000 s^−1^, see kinetic scheme in Fig. 1) as measured by Schneggenburger and Neher [15] and made this rate equal for both the synchronous and asynchronous pathways. This is consistent with observations which showed that slow-to-release vesicles have the same release transients [48] as other vesicles, when calcium was un-caged so that calcium concentration was uniform across the presynaptic terminal of the calyx. This suggests equal neurotransmitter fusion release rates,*γ*, since in calcium-uncaging protocols, it is likely that calcium ion binding is not the rate-limiting quantity.

However, it is only possible to fit all the release data for CA3-CA1 synapses when we set the value of the neurotransmitter fusion rate, *γ* , to be 40 times slower for the asynchronous pathway relative to the synchronous pathway, assuming that vesicles act independently aside from the refractory period. An alternative possibility is that there might be additional coupling in the active zone beyond the refractoriness, coupling that makes the active zone behave as if there were a single gate. This suggestion comes from our simulations with a phenomenological model (mentioned earlier) of the entire active zone where the spurious early peak in asynchronous release is eliminated without having to change the vesicle fusion rates. The overall effect of this inhibitory coupling is to reduce the effective asynchronous neurotransmitter fusion rate. Developing this possibility further would require a better understanding of the proteins that are responsible for the coupling and including the concomitant explicit sensor-sensor coupling in the kinetic scheme. Experimentally, one would need to develop knock-outs of the coupling proteins and test these for evidence of enhanced asynchronous release rates, especially the existence of an early release peak not present in wild-type synapses.

Finally, our study is built upon an underlying assumption that spontaneous release, synchronous release and asynchronous release take place from the same RRP [40], [62]. This has been questioned recently [63], [64]. We do not explicitly address any alternate possibilities in this present study.

## Materials and Methods

Simulations were performed using MCell, version 3 [65], [66]. MCell uses Monte Carlo algorithms to simulate volume and surface reaction-diffusion of discrete molecules in complex spatial environments with realistic cellular and sub-cellular geometry. This allows for detailed study of the effect of the spatial organization and stochastic reaction-diffusion dynamics on the temporal evolution of key system variables. We modelled a 0.5 µm×0.5 µm×4 µm volume of simplified en passant axon segment with physiologic spatial distributions and concentrations of ligands and molecules. Initial concentrations, locations, diffusions constants, and rates and their sources used for the MCell model are specified in Table 1. Further validation of the parameters used comes from the shape and amplitude of the calcium response to action potential in our simulations which is consistent with experimental data [8], [9].

**Figure 10:**
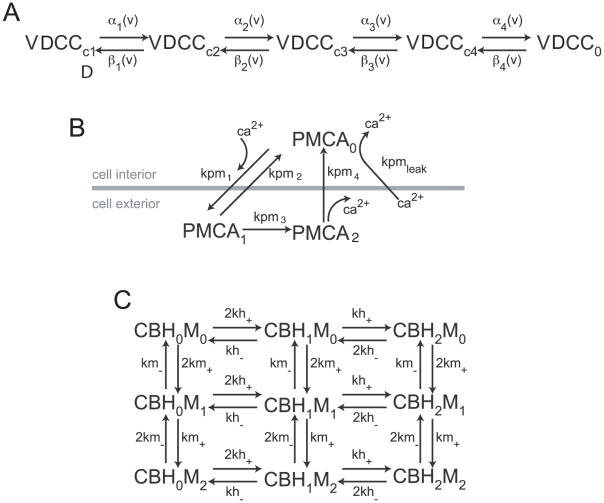
Kinetic schemes. (**A**) Voltage Gated Calcium Channels (**B**) PMCA pump and (**C**) Calbindin. The rates with the respective references appear in Table 1.

The apparent diffusion constant of calcium, a key parameter for physiological relevance of our results, was matched in the model to the measured value (50 µm^2^/Sec) [14]. This value is substantially slower than the initial cytoplasmic free diffusion constant of 220 µm^2^/sec specified for the simulation and arises because our model has an accurate description of the calcium binding kinetics of mobile calcium binding proteins in the synapse (See Fig. 10 for kinetic schemes). The calcium concentration was clamped at 100 nM at both ends of the axon segment. The simulation time step for calcium was specified to be 0.1 µsec and for all other molecules was 1.0 µsec. The release transients presented in the figures is a result of N = 10000 simulations for each parameter set. For our stochastic simulations the standard deviation of the vesicular release number is √r where r is the total number of release events observed in a temporal bin, t_b_ (t_b_  = 10 ms or 1 ms ). The value of ‘r’ in every bin can be determined by r = release rate . N . t_b_ . The docked vesicles were clustered in a hexagonal array with largest center-to-center distance between vesicles of 35 nm.

## Supporting Information

Text S1 Supporting information.
(0.23 MB PDF)
