# Supplementary material for: Modelling Vesicular Release at Hippocampal Synapses
Source: PLoS Comput Biol. 2010 Nov 11;6(11):e1000983. doi: 10.1371/journal.pcbi.1000983 (PMC2978677; doi:10.1371/journal.pcbi.1000983)
Supplement: Text S1 — Supporting information. (0.23 MB PDF) [file pcbi.1000983.s001.pdf]

Figure 1

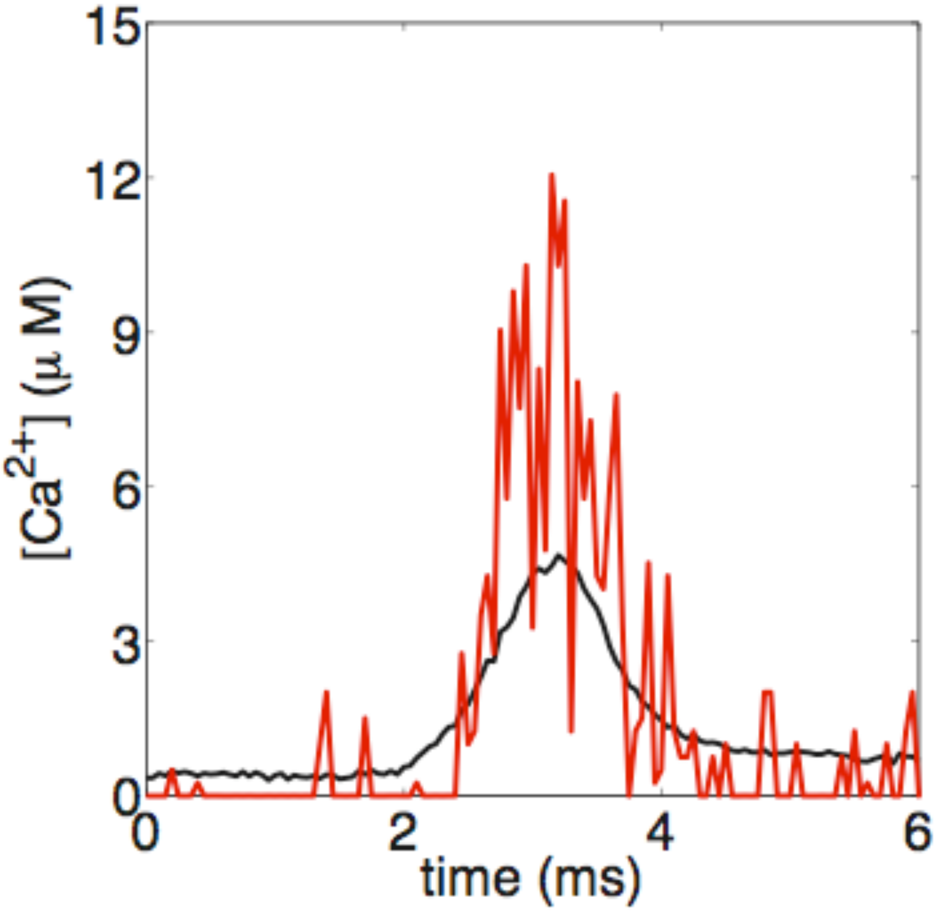

Fig. 1 Sample calcium transient. Single run (red) and average of 1000 trials (black) for release probability of 20% generated by 48 channels at 250 nm from the active zone. The calcium is measured 10 nm above the active zone.

Figure 2

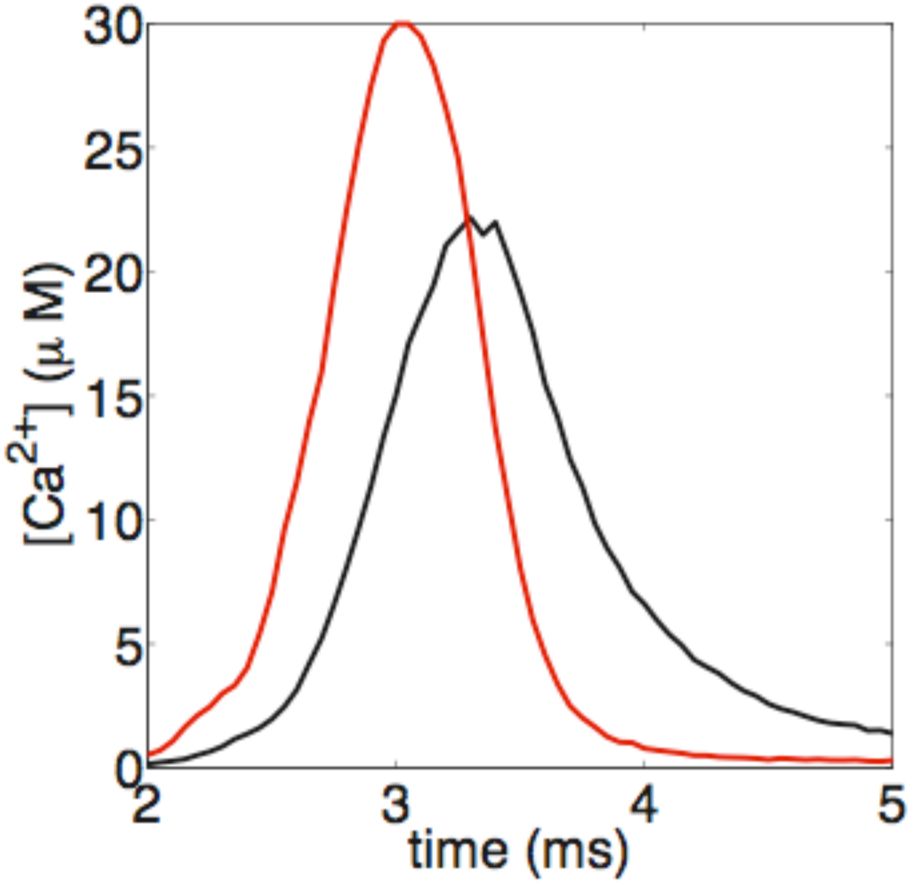

Fig. 2 Sample local calcium transients for different distances. The calcium transient generated by 32 VGCCs placed at 100 nm (red) and 160 VGCCs at 400 nm (black) lead to the same release probability of ~0.9. Ultrasynaptic structure such as the number and placement of the channels with respect to the calcium sensor modulate the shape of the calcium local calcium transient leading to non-overlapping neurotransmitter response curves for various l_c seen in Fig. 2B.

Figure 3

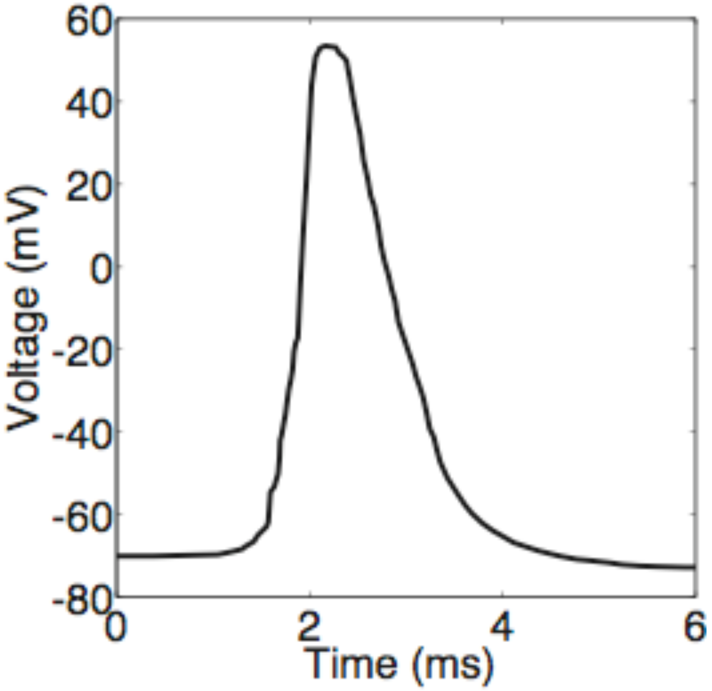

Fig. 3 Boutonic action potential waveform used in the simulations to initiate calcium response from the VDCCs. (Bischofberger et. al, Journal of NeuroSci. 2002)

Figure 4

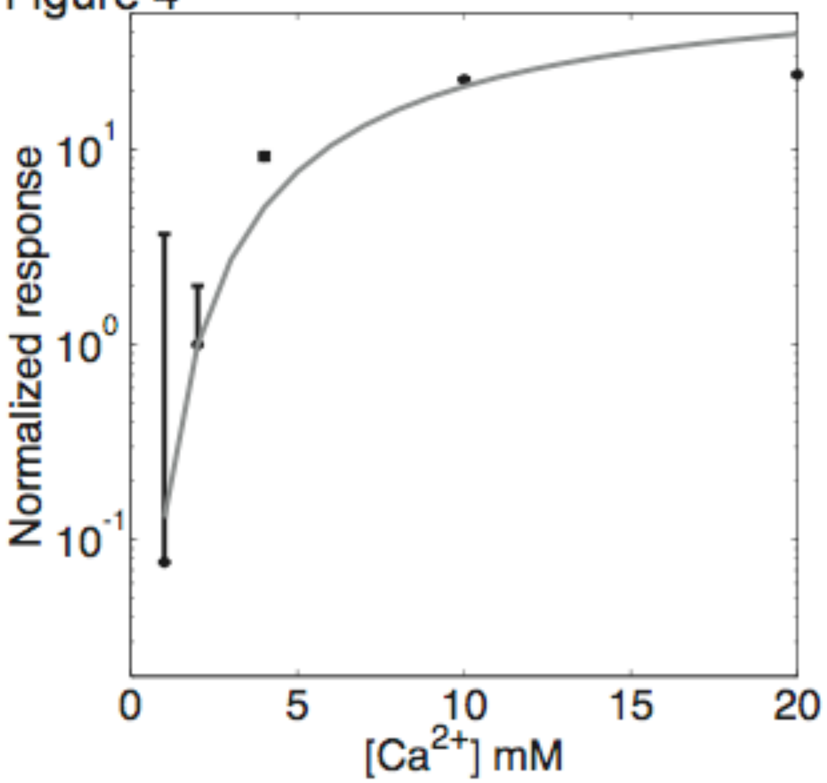

Figure 4 Fit to Dodge and Rahaminoff equation. Simulated effects of varying the extracellular calcium concentration on the number of vesicles released in the first 20ms for direct comparison with Goda and Stevens (1994, Figure 3). The results fit well with Dodge and Rahamimoff equations with an exponent of 4. The data was fitted to $\left[\frac{a_0[Ca^{2+}]}{1+a_1[Ca^{2+}]}\right]^4$, with a_0=0.75 and a_1= 0.25. Thus the apparent cooperativity is ~4 even though there are 5 binding sites.

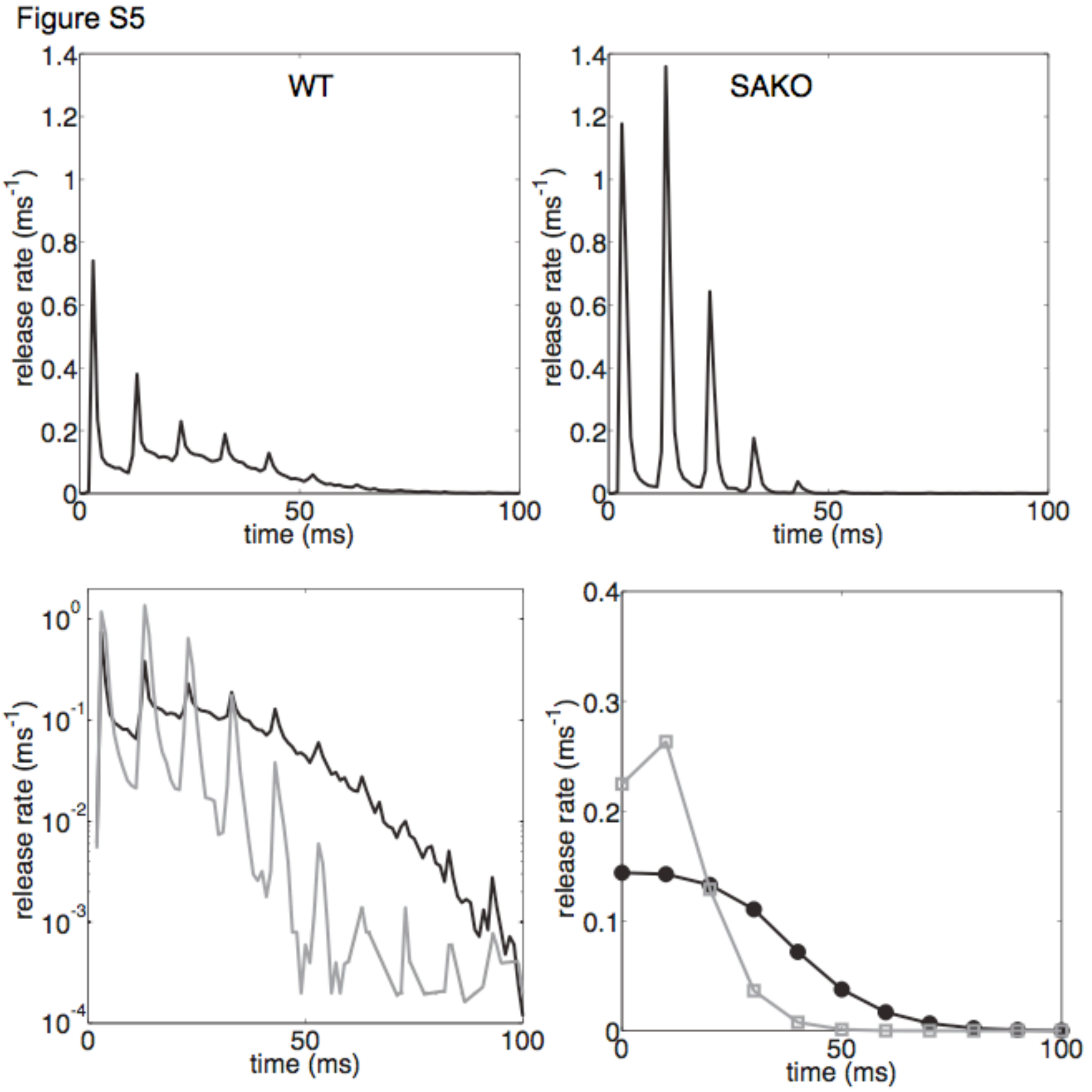

Fig. 5 Response to 100 Hz train stimuli for a high release probability synapse (p_r=0.9). Release rate for wild type (**A**) and asynchronous release sensor KO (**B**) plotted in 1 ms bins. The same data is plotted on a log scale to show higher base level release (black line) due to the presence of asynchronous sensor in the wild type (black line **C**). The grey line in (**C**) is asynchronous sensor KO. Here the effect of including the asynchronous sensor is completely opposite to simulations carried out for low frequency stimulus (10 Hz) in Fig. 8. For this fast stimulus the asynchronous release does not build up enough to benefit the subsequent incoming stimulus. The peak release rate for wild type (**A**) remains lower than peak release rate of the KO. This is because the forward binding rate of asynchronous pathway is fast enough to compete for the incoming

calcium ions but it is too slow to release (due to much slower fusion rate). This is also leads to overall reduction in release rate in the wild type as seen in (**D**). Data binned in 10 ms bins for each stimuli is shown here (wild type – black line, asynchronous KO – grey line).
